# Meso fracture characteristics of granite and instability evolution law of surrounding rock in deep cavern

**DOI:** 10.1038/s41598-022-06833-0

**Published:** 2022-02-22

**Authors:** Yunhe Ao, Chuang Sun, Baoxin Jia, Jianjun Zhang

**Affiliations:** School of Civil Engineering, Liaoning Technical University, Fuxin, 123000 Liaoning People’s Republic of China

**Keywords:** Civil engineering, Theory and computation

## Abstract

In order to analyze the influence of meso-structure and meso-parameters on deep granite characteristics, a construction method of variable radius proportional clump model was proposed with particle flow method. The clump particle flow structure was constructed which suited the mechanical characteristics of granite. The deep cavern numerical calculation model of gradual particle density was constructed using the variable radius proportional clump model construction method, and the macroscopic fracture law of deep cavern surrounding rock was analyzed. The results show that meso parameters have lower effects on the compressive and tensile ratios of particle structures in the ball and clump models. It is also found that clump structure is greatly influenced by particle proportion and size while ball model is only slightly influenced by particle size. Furthermore, the compressive and tensile strength curves and fracture modes of numerical simulations and laboratory tests are in good agreement. In addition, the calculated results of the tunnel after simulated excavation are very close to the engineering practice. There are obvious shear failure areas on the right vault and left wall of the tunnel, and the shape and depth of the fracture area are basically the same.

## Introduction

**Figure 1:**
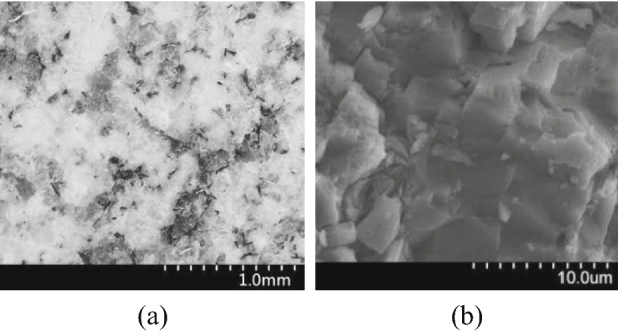
Meso and micro properties of granite. (**a**) Mesoscopic features, (**b**) microscopic features.

Rocks in nature are exposed to complex geological environments and long-term geological tectonic effects with discontinuous and heterogeneous complexities^1–3^. Geotechnical engineering calculations are generally calculated using discrete element method to evaluate rock mass mechanical properties. Due to the great advances in the past years, discrete element method has realized the simulation analysis of the closure, generation and extension of microcracks in brittle rocks. The particle flow method in discrete element method is the main simulation methods for rock fracture in laboratory, engineering and geological scales^4–6^. Mesoscopic and microscopic rock observations have shown that most rocks are consisted of tightly combined irregular mineral particles. Mesoscopic and microscopic structures of granite are shown in Fig. 1. Mineral particle shape affects the macroscopic mechanical behaviors of rocks. Hence, applying discrete element method for constructing numerical calculation model according to the mechanical properties of rocks and achieving mesoscopic parameters for particle flow model is critical in investigation of microscopic fracture properties of rock masses and analysis of macroscopic failure law of deep cavern surrounding rock.

Recently, rock meso-fracture has been studied in terms of fracture propagation by several researchers using particle flow method. For instance, Lin et al.^7^ conducted failure tests on marble during unloading confining-pressure under constant axial stress and simulations with the particle flow code and studied the influence mechanism of the unloading rate of the confining pressure, initial unloading stress, and confining pressure on the failure characteristics of marble. The field evolution, cracking process and mechanical properties of stress-containing defected composites were investigated by Liu et al. by laboratory tests and numerical simulations^8^. The influence of particle bonding on rock fracture properties was studied by Scholtes et al.^9^ using discrete element method. Bahrani et al.^10^ developed SDA method for the estimation of microcracked rock strength and ultimate crack density in a variety of rock types. Shi et al.^11^ investigated the composition ratios of different granite samples and evaluated the influences of different microfracture distributions and mineral ratios on the macroscopic mechanical characteristics of rock samples using discrete element method. Xu et al.^12^ discussed the influence of joint inclination of Phyllite failure mode and studied the progressive failure process of Phyllite.

In terms of numerical calculation research on engineering scale, scholars have also carried out extensive research. For instance, Abierdi et al.^13^ compared and analyzed by indoor model test and numerical simulation method based on particle flow program PFC^2D^, revealed the changes of microscopic stress and force chain in the process of crack propagation and loading and unloading, and revealed the variation law of radial stress and tangential stress of surrounding rock in crushed zone, as well as the influence of initial stress on failure load. Based on the trial-and-error method, Peng et al.^14^ first proposed a parameter test method for a large model, and then tested the method by a large tunnel model test. The research results showed that the parameters checked by a small model directly could improve the mechanical strength of the large model, and also had a great impact on the failure mode. Li et al.^15^ verified the theoretical analysis by using a two-dimensional numerical model established by particle flow program, further studied the failure characteristics of the existing tunnel under the coupling action of static in-situ stress and dynamic unloading wave, and discussed the effects of unloading rate, unloading path, in-situ stress and excavation radius on the dynamic response of the existing tunnel.

To sum up, scholars from all over the world have carried out extensive research on numerical simulation of rocks using PFC particle flow program, but the research on efficient modeling method is rarely reported. In this paper, a construction method of variable radius proportional clump structure was developed. The influences of meso-structure and meso-parameters on the compressive and tensile properties of simulated rocks were investigated. The meso fracture characteristics of different cracked granite samples were evaluated and clump particle flow structures were constructed based on the mechanical characteristics of granite specimens. Meso mechanical parameters and laboratory test results were compared to verify the reliability of variable radius proportional clump structure and meso mechanical parameters. A deep cavern particle flow model with gradual particle density was constructed and the local fracture law of deep surrounding rocks was investigated to analyze the local instability law of surrounding rock under high stress condition. Our findings can provide significant guidelines for deep engineering design and monitoring research.

## A clump structure with variable radius ratios and parameters

### Basic principle of particle flow code

**Figure 2:**
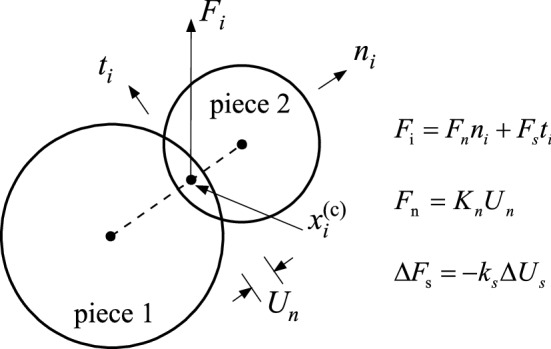
Diagram of two contacting particles.

Particle flow code (PFC) is a discrete element method which can be applied for the simulation of the interaction and motion of a circular particle medium. The basic mechanical characteristics of the media were considered in terms of basic particle structures of media. The difference of continuum mechanics and PFC was that particle contact states strongly affected the basic properties of a certain medium under various stress conditions. Discrete bodies are considered as collections of finite discrete particles and the body is ideally assumed as a group of contacting particles being independent but in interaction, as shown in Fig. 2.

where *x*_*i*_^(c)^ is overlapping region center along the direction of the joining line of the centers of particles, *F*_*i*_ is contact force vector, *t*_*i*_ and *n*_*i*_ are unit vectors describing contact plane, and *F*_*s*_ and *F*_*n*_ are shear and normal force components, respectively. *U*_*n*_ is the overlap and *k*_*n*_ denotes the normal stiffness of contact, Δ*F*_*s*_ is elastic shear force increment, *k*_*s*_ is the shear stiffness of contact, and Δ*U*_*s*_ denotes relative shear–displacement increment.

In particle flow method, large deformation processes of different materials are generally investigated from linear elastic stage to fracture failure directly reflecting crack formation, propagation and penetration. Therefore, this method is the most commonly used method in the simulation of micromechanical and macroscopic rock behaviors.

### Parallel bond model and clump structure

Contact bond (CB) and parallel bond (PB) models are developed in PFC. Contact bond model can be considered as two elastic springs having constant shear and normal stiffnesses at a given point. However, parallel bond model approximates particle pair bonding as cement material, assuming elastic interactions between particles parallel to sliding or contact bonding constitutive model^16^.

**Figure 3:**
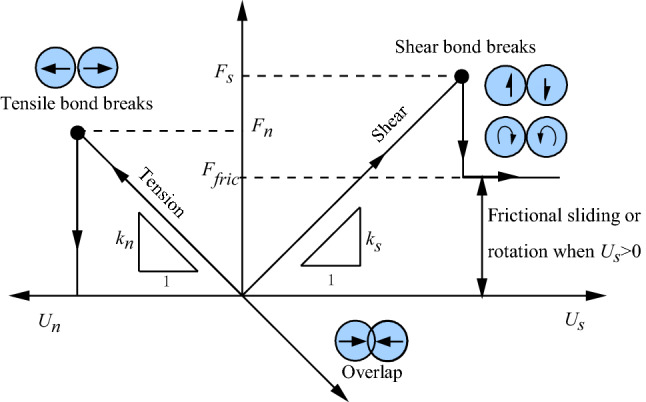
Tangential and normal mechanical responses of CBM.

Particles in PFC are free to move in normal and shear directions and can also rotate between particles. This rotation may induce a moment between particles. Contact bond model is not able to resist this moment as long as particles are in contact, even after bond breakage. Strength is decreased to a constant value which is determined by friction and the contact stiffness still exists. After bond breakage, however, bond tensile strength is zero regardless of the contact status of particles, as shown in Fig. 3.

**Figure 4:**
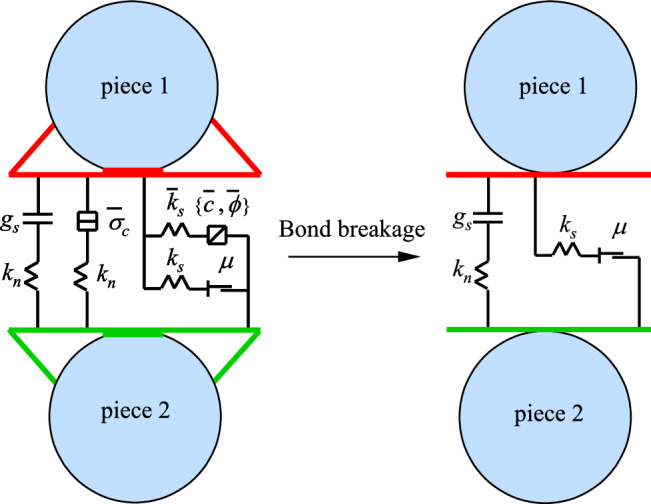
Diagram of parallel bond model (PBM).

PB model is consisted of linear and bond models. Linear model is not able to resist relative rotations. Tangential slip is mainly determined by friction. Bond model is able to resist moment and force and has tangential shear and tensile strengths. Based on Mohr–Coulomb criterion, parallel bond model acts similar to linear model at too high loadings when bonds are broken, as shown in Fig. 4.

where *k*_*n*_ and *k*_*s*_ are normal and shear stiffness, respectively, *c* is cohesion, *g*_*S*_ is bond surface gap, $\overline{\sigma }_{c}$ is tensile strength, $\overline{\mu }$ is friction coefficient and *ϕ* is friction angle.

The values of contact and bond stiffness determine parallel bond model stiffness. The difference of parallel and contact bond models is that, bond fracture immediately reduces stiffness in parallel bond model, which not only affects bonded particle stiffness, but also influences the whole model macro stiffness. Therefore, parallel bond model is found to be more appropriate for hard materials such as rock and was applied for the simulation of the micro and macro fracture characteristics of granite.

**Figure 5:**
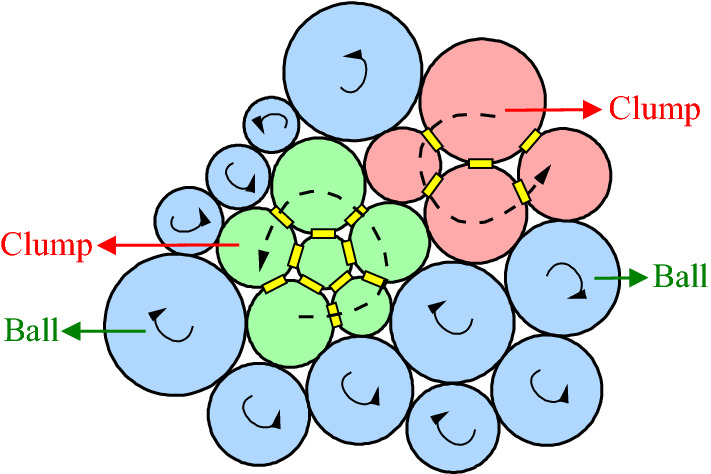
Ball and clump rotation mechanisms in particle model.

Cho et al.^17^ developed clump parallel bonding model (CPBM) by combining several particles for developing clump structure. In the model, particles are considered as individual rigid bodies and agglomerated particles can overlap as a deformed body to any degree, which do not break no matter how much force acts on them. Therefore, agglomerated particles behave as individual particles with irregular shapes moving as rigid bodies. Particle rotation speeds in clumps are fixed. It can be seen from Fig. 5 that only the clump itself has rotation speed. Potyondy et al.^18^ showed that traditional PFC model greatly deviated from experimental findings. However, clump modeling method can limit particle rotation and simulate particle loading and failure processes.

### Construction of clump model with variable radius ratios

Nowadays, three major methods are available clump modeling which are described below. Cho et al.^17^ developed an irregular particle model by limiting clump particle number. Yoon et al.^19^ established a concentric particle clump by arranging particles in successive circles with particles located outside the circle forming a group. Shi et al.^20^ developed an irregular arranged the outer contours of the clumps by several overlapping pebbles.

**Figure 6:**
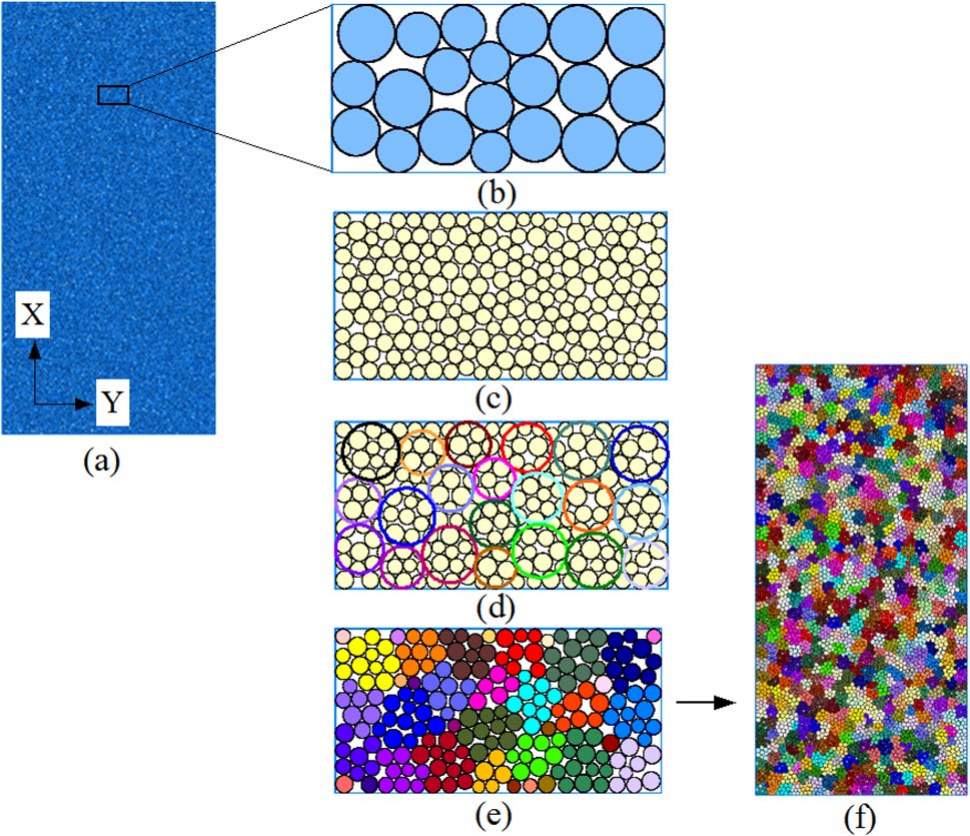
Diagram of clump modeling method.

In this paper, we proposed a clump structure with variable radius ratios based on particle flow principle. The following modeling steps were taken:

1. Large particles (Ball_max_) with radius ratio *R*_min_:*R*_max_ = 1:*x* (*x* > 1) were generated in the given wall (Wall), as shown in Fig. 6a. A small rectangle in the model was taken as an example to enlarge, as shown in Fig. 6b. Data such as particle radii and coordinate points were written in a database by Fish language. Then, these large particles (Ball_max_) were deleted.
2. Small particles (Ball_min_) of *r*_min_:*r*_max_ = 1:*y* (*r* < *R*, *y* > 1) were regenerated in certain wall ranges, as shown in Fig. 6c.
3. As shown in Fig. 6d, coordinate and radius values of large particles were restored from the database and small particles were marked in the wall (Wall) which arranging them in circular shapes into groups (Group) around large particles. Then, unlabeled but contacting particles are grouped using Fish language. The number of such generated groups determined the number of clumps that subsequently established.
4. Each small particle group was converted into a clump template using Fish language and clump structure was constructed based on these templates. Finally, these small particles (Ball_min_) were deleted, as shown in Fig. 6e. The final structural model is shown in Fig. 6f.

In the clump structures constructed based on the above method, clump size ratios could be freely adjusted by setting pebble radius. Macroscopic structural mechanical characteristics of particle flow model were adjusted by setting these structural dimensions.

### Clump model parameters

Using variable radius proportional clump model developed in this work, Brazilian disc splitting and uniaxial compression tests were simulated by particle flow program PFC^2D^ to evaluate the effects of meso parameters and particle sizes of clump and ball models on simulated rock macro mechanical characteristics and it was observed that the obtained results complied well with laboratory findings. The uniaxial compression test particle flow test specimen had length 100 mm and width 50 mm. Brazilian disc split test specimen diameter was 50 mm and small particle (Ball_min_) in the model had the following dimensions *r*_min_ = 0.26 mm, *r*_min_:*r*_max_ = 1:1.5.

#### Particle size effects

**Figure 7:**
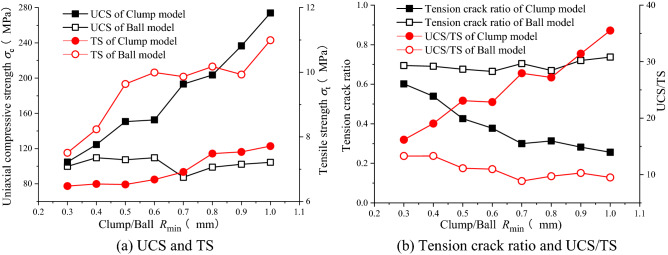
The effects of clump/ball particle size on macroscopic mechanical parameters of specimens. (**a**) UCS and TS, (**b**) tension crack ratio and UCS/TS.

As given in Table 1, meso-mechanical parameter values and particle size ratio of large particles were kept constant at *R*_min_:*R*_max_ = 1:1.5 in the proposed model. Minimum particle sizes *R*_min_ of clump and ball structures were changed in the range of 0.3–1.0 mm. Clump and ball size effects on macroscopic mechanical characteristics of the simulated rocks were also investigated. Figure 7 shows the obtained simulation results.

**Table 1:** Model mechanical parameters.

Parameters	Ball model/clump model
Particle stiffness ratio *k*_*n*_*/k*_*s*_	2.0
Particle friction coefficient *µ*	0.2
Parallel bond modulus E*/Gpa	2.0
Parallel bond stiffness ratio *k*_n_*/k_s_*	2.0
Parallel bond tensile strength *σ*_*b*_/Mpa	36
Parallel bond cohesion *c*_*b*_/Mpa	27
Friction angle *ƒ*/°	32

Figure 7a shows the variations of uniaxial compressive strengths (UCS) and tensile strengths (TS). Since clump structure restricted particle rotation, the compressive strength of clump model was higher than that of ball model. By increasing clump radius, particle numbers of clump structure and boundary friction effect between clump structures were increased. Hence, uniaxial tensile and compressive strengths of clump model specimens were continuously increased with increasing clump radius. However, increasing of ball radius did not significantly change uniaxial compressive strength of ball model specimens but increased their tensile strength.

Figure 7b shows the ratio of uniaxial compressive strength to tensile strength (UCS/TS) as well as tensile crack ratios of clump and ball models. It was seen that the tension crack ratios of clump model specimens were decreased as radius was increased. The reason for this was that, by the propagation of tensile crack along axial direction, the structural properties of clump deviated stress path from maximum principal stress direction resulting in shear failure. The influence of the variation of particle size on the propagation path microcracks in ball model specimen was not clear and tensile crack ratio was stabilized. The UCS/TS of clump model specimens was significantly increased as radius was increased, while that of ball model specimens was only slightly influenced by particle radius.

#### Mechanical parameter values effects

Radius ratio of *R*_min_:*R*_max_ = 1:1.5 was adopted for clump and ball models. Using meso-mechanical parameter values given in Table 1, the value of one parameter was changed keeping constant the remaining parameters. In similar parameter variation ranges, the influences of parallel bond stiffness ratio *k*_n_^*^/k_s_^*^, parallel bond modulus E^*^, parallel bond cohesion *c*_*b*_ and parallel bond tensile strength *σ*_*b*_ on uniaxial compressive tensile and strengths as well as UCS/TS were evaluated.

**Figure 8:**
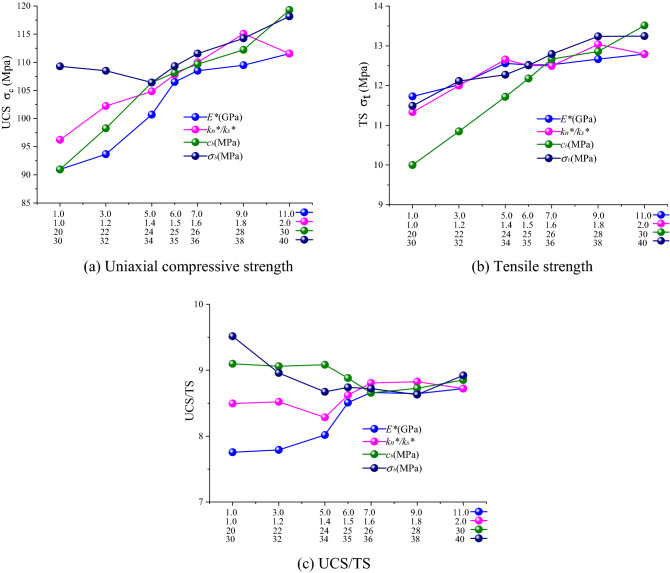
The effects of meso parameters on macro mechanical parameters of ball model specimens. (**a**) Uniaxial compressive strength, (**b**) Tensile strength, (**c**) UCS/TS.

Simulation results obtained for ball model are shown in Fig. 8. By the increase of parameter values, uniaxial compressive and tensile strengths were increased and UCS/TS was stabilized. After the calculation of parameter variations of ball model, it was revealed that each parameter had a certain effect on UCS/TS with the effect of parallel bond modulus E^*^ being the strongest. However, after a certain parameter value was reached, its effect on UCS/TS was decreased; this was very obvious when parallel bond stiffness ratio *k*_n_^*^/k_s_^*^ of 1.6 was achieved.

**Figure 9:**
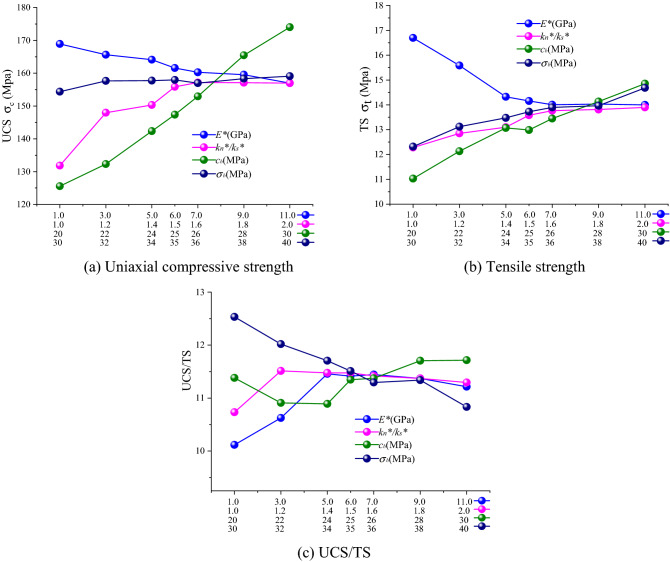
The effects of meso parameters on macro mechanical parameters of clump model specimens. (**a**) Uniaxial compressive strength, (**b**) tensile strength, (**c**) UCS/TS.

Clump model simulation results are shown in Fig. 9. The parallel bond cohesion *c*_*b*_ and parallel bond tensile strength *σ*_*b*_ obviously affected the UCS/TS of clump model with parallel bond tensile strength *σ*_*b*_ having the strongest effect. By increasing the parallel bond tensile strength *σ*_*b*_, UCS/TS was decreased. Parallel bond stiffness ratio *k*_n_^*^/k_s_^*^ did not have significant effect on clump model UCS/TS. However, it had a certain effect on model fracture mode.

Parameter comparison revealed that all parameters had certain effects on rock macroscopic mechanical properties. However, it was observed that UCS/TS changes were not obvious with the parameter within a certain parameter range by comparing the Ball model with the Clump model. It was also witnessed that particle size and shape had the strongest effect on rock mechanical properties under compression and tension while constructing particle flow models for various rocks (such as shale, mudstone and sandstone) while model mechanical parameters can only play a role of fine adjustment.

## Meso fracture characteristics of granite

### Mechanical properties of granite

**Figure 10:**
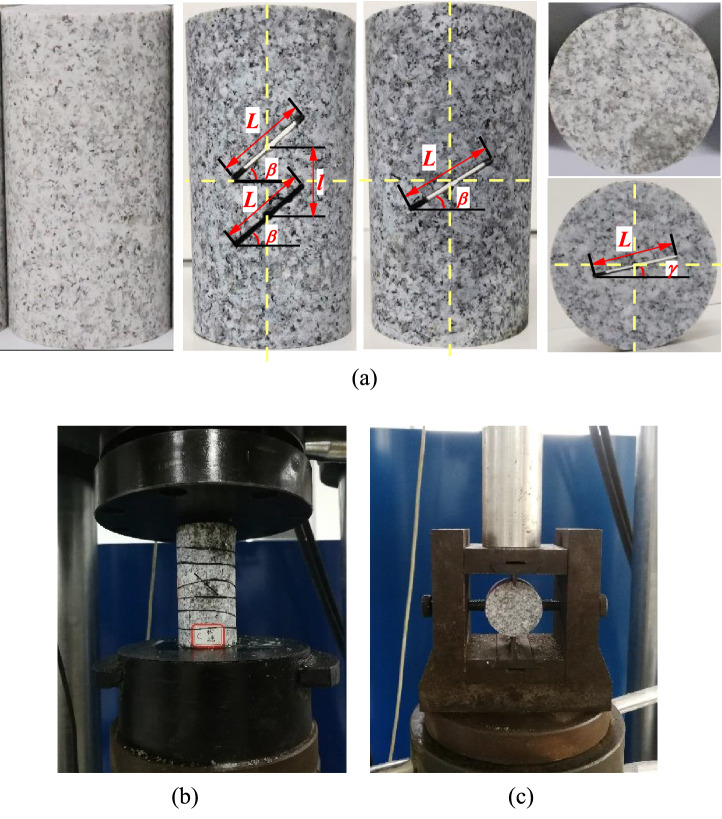
Granite samples and laboratory test conditions, (**a**) granite samples, (**b**) uniaxial compression test, (**c**) Brazilian splitting test.

Granite samples were collected from − 1080 m horizontal working face of Xincheng Gold Mine in Shandong Province. Rock masses were uniform with good overall integrity. Test specimens were processed into φ50 mm × 100 mm cylindrical and φ50 mm × 25 mm disc specimens, and the center of fracture specimens were hydraulically cut on the basis of the complete specimen to obtain cracked granite specimens. Type A cylindrical specimens had double cracks and type B cylindrical specimens had single cracks. The crack length of the uniaxial cylinder specimen and the Brazilian disk specimen was 25 mm. The angle between the crack and the horizontal direction of the cylinder and disk specimens is *β* and *γ*. The accuracy of machining met the recommended test specifications of International Rock Mechanics Society. The uniaxial compression and Brazilian splitting tests were performed on a GAW-2000 microcomputer controlled electro-hydraulic servo rigid pressure tester (Chaoyang Test Instrument Co., Ltd). Granite samples and laboratory test conditions are presented in Fig. 10. Mechanical parameters of specimens were calculated, as summarized in Table 2.

**Table 2:** Mechanical parameters of specimens.

Uniaxial compression test			Brazilian splitting test		
Type	Crack angle *β*/°	UCS *σ*_*c*_/MPa	Type	Crack angle *γ*/°	TS *σ*_*t*_/MPa
N	–	138.73	S-N	–	11.85
A-35	35	48.03	S-0	0	4.97
A-45	45	60.68	S-40	40	4.07
B-35	35	47.87	S-60	60	2.27
B-45	45	56.58	S-90	90	3.11

### Calibration of particle flow model parameters

**Figure 11:**
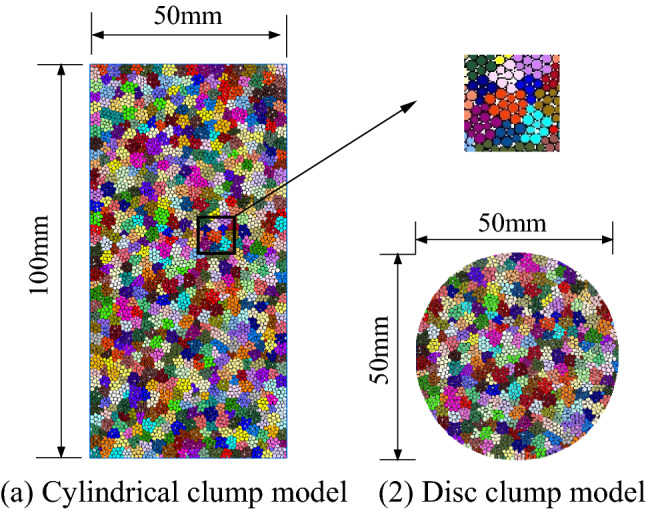
Diagram of clump model. (**a**) Cylindrical clump model, (**b**) disc clump model.

Numerical simulation of Brazilian disc splitting and uniaxial compression test were conducted by particle flow PFC^2D^ software using variable radius proportional clump model. Numerical calculation model size was the same as that of laboratory test. Figure 11 shows the schematic diagram of clump model. Pebble numbers of uniaxial and Brazilian clump models were 14,616 and 4986, respectively. During parameter calibration, granite particle flow model was adjusted according to the particle size and mechanical parameter effects on UCS and TS. Based on the laboratory results of granite compressive and tensile strengths, clump *R*_*min*_ was determined to be 0.6 mm. Mechanical parameter values in Table 1 were fine-tuned by taking the impacts of cohesion *c*_*b*_ and tensile strength *σ*_*b*_ on clump model UCS/TS and parallel bond stiffness ratio *k*_n_^*^/k_s_^*^ on fracture mode into consideration. Model mechanical parameters were calculated as given in Table 3.

**Table 3:** Model mechanical parameters.

Parameters	Value	Parameters	Value
Ball *R*_min_/mm	0.26	Parallel bond modulus E*/Gpa	2.0
Ball radius ratio *R*_min_:*R*_max_	1.5	Particle stiffness ratio *k*_n_*/k_s_*	2.5
Ball stiffness ratio *k*_*n*_*/k*_*s*_	2.0	Parallel bond tensile strength *σ*_*b*_/Mpa	32
Ball friction coefficient *µ*	0.2	Parallel bond cohesion *c*_*b*_/Mpa	24
Clump *R*_min_/mm	0.6	Particle friction coefficient µ*	0.2
Clump radius ratio *R*_min_:*R*_max_	1.5	Friction angle *ƒ*/°	32

### Uniaxial compression test results

The stress–strain curves and final fracture modes of uniaxial compression laboratory test and numerical simulation are summarized in Tables 4 and 5.

**Table 4:**
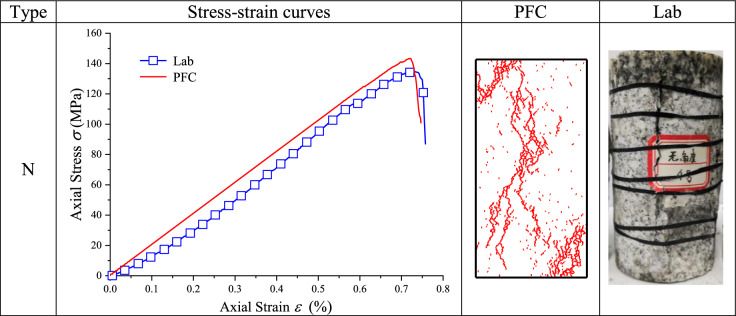
Uniaxial compression test results of type N granite specimen.

**Table 5:**
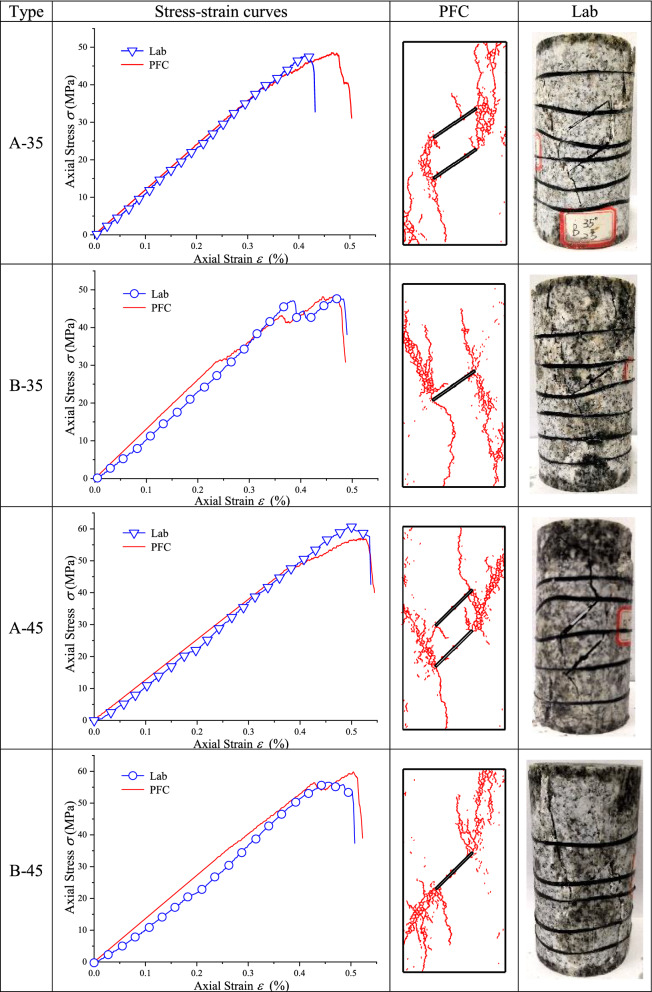
Uniaxial compression test results of cracked granite specimens.

Table 4 illustrates that the numerical simulation stress–strain curve of type N granite specimen agreed well with laboratory test stress–strain curve. The uniaxial compressive strength of laboratory test was 135.86 MPa and that of numerical simulation was 141.39 MPa, with a relative error of 4.07%. The uniaxial peak strain was 0.76% in laboratory test and 0.72% in numerical simulation, with a relative error of 5.26%. The elastic modulus of laboratory test was 19.88 GPa, and that of numerical simulation was 20.47 GPa, with a relative error of 2.91%. At the same time, axial stress was rapidly decreased after a peak value and samples lost their stability and exhibited typical brittle properties. The fracture mode of numerical simulations was splitting along loading axis, which complied with laboratory result.

Table 5 shows that the numerical simulation stress–strain curves agreed well with laboratory test stress–strain curves of type B-35, type A-45 and type B-45 cracked specimens. As for type A-35 cracked specimen, the uniaxial peak strain of laboratory test was 0.42%, the peak strain of numerical simulation was 0.47%, with a relative error of 11.90%. For type A-35, type B-35 and type A-45 cracked specimens, the crack propagation mode of numerical simulation was very close to that of the laboratory test. However, for type B-45 cracked specimen, the propagation direction of the crack along the end point of the prefabricated crack in laboratory test and numerical simulation was opposite, indicating that the agreement between the two was general.

### Brazilian disc splitting test results and analysis

The stress–strain curves and final fracture modes of Brazilian disc splitting test and numerical simulation are summarized in Tables 6 and 7.

**Table 6:**
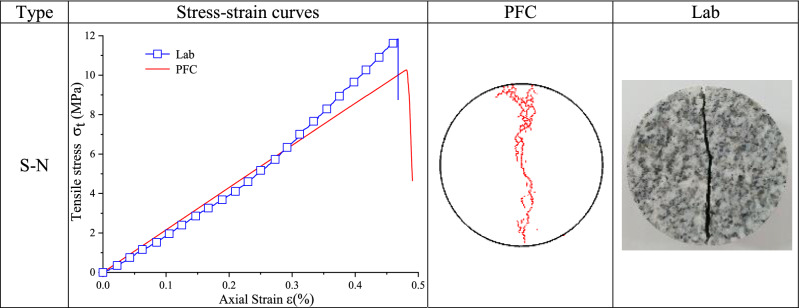
Brazilian splitting test results of type S–N granite specimen.

**Table 7:**
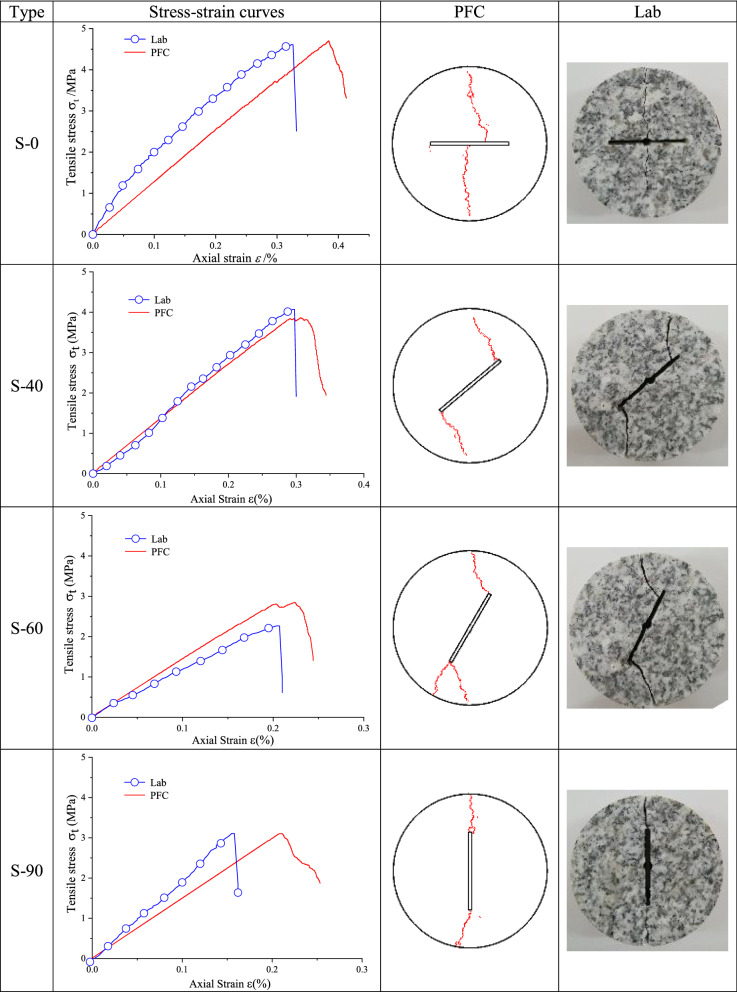
Brazilian splitting test results of cracked granite specimens.

Table 6 illustrates that the simulation stress–strain curve of type S–N cracked granite specimen agreed well with laboratory test stress–strain curve. Laboratory and simulation tensile strength values were 11.85 MPa and 10.37 MPa, with a relative error of 12.49%. The uniaxial peak strain of laboratory test was 0.47% and the peak strain of numerical simulation was 0.48%, with a relative error of 2.13%. The elastic modulus of laboratory test was 2.66 GPa and that of numerical simulation was 2.25 GPa, with a relative error of 15.41%. At the same time, simulation fracture mode was tensile failure along loading axis, and the final fracture mode was in good agreement with the laboratory test result.

Table 7 shows that a good compliance was between simulation and laboratory curves and fracture modes of type S-40 and type S-60 cracked granite disc specimens. There was a slight deviation between numerical calculation curve and laboratory test curve of type S-0 cracked specimen. The uniaxial peak strain of laboratory test was 0.33% and the peak strain of numerical simulation was 0.38%, with a relative error of 15.15%. The cracks in numerical simulation propagated from the center of the prefabricated crack down to the end of the specimen and from a position about 6 mm away from the center of the prefabricated crack and expanded upward to the end of the specimen. The crack in the laboratory test started from the center of the prefabricated crack and extended up and down to the end of the specimen. As for type S-90 cracked specimen, the uniaxial peak strain of laboratory test was 0.16% and the peak strain of numerical simulation was 0.21%, with a relative error of 31.25%. The elastic modulus of laboratory test was 1.08 GPa and that of numerical simulation was 0.96 GPa, with a relative error of 11.11%. The fracture mode of the numerical simulation was generally consistent with that of the laboratory test.

## Evolution law of local rupture and instability of surrounding rock in deep cavern

The fracture problem of surrounding rock under high stress has always been emphasized by geotechnical engineering scholars^21,22^. Especially in the research of hydraulic fracturing and rock burst, and scholars are still searching for new methods to reveal the fracture law.

Many excellent results have been achieved concerning the damage mechanisms of circular tunnel caverns under different geological conditions. For example, Diederichs^23^ applied a composite robust field yielding model to nonlinear modeling of support design using a spalling limit to differentiate stress paths that lead to crack propagation and spalling from those that incur stable microdamage prior to conventional shear failure at higher relative confinements. Farahmand and Diederichs^24^ investigated the evolution of damage and pore pressure around two unsupported circular granite tunnels by using universal distinct element code (UDEC) to verify the validity of the effective stress law for low porosity rock drainage peak strength by a series of biaxial compression test simulations of the Terzaghi. Martin et al.^25^ found that the rock damage criterion using friction parameters significantly underestimated the depth of brittle damage, while the use of brittle parameters agreed well with field observations, and the Hoek–Brown brittle parameters could be used to estimate the depth of brittle damage around the tunnel, the support demand load due to stress-induced damage, and the optimal geometry of the opening. Cai et al.^26^ proposed a damage characterization method based on microseismic event monitoring of rock masses near excavations and a damage-driven numerical model with microseismic data as input and damage states described by fracture density. Naji et al.^27^ used an empirical method to evaluate the propensity of occurrence of impact ground pressure, and the actual damage area of deep excavation was better predicted by FLAC^2D^ numerical simulation.

In view of the above analysis, it is of great scientific importance to construct a numerical calculation model that conforms to the mechanical properties of the rock and to analyze the evolution of instability in deep caverns. The analyses presented in this study focuses on the construction of an efficient and accurate engineering-scale numerical calculation model based on the variable radius scale clump model construction method and the selected mechanical parameters to simulate the mechanical response of the surrounding rock after excavation of the horseshoe roadway. Therefore, whether the model based on clump particles can realistically replicate the local instability evolution law of the high-stress horseshoe roadway surrounding rock after excavation is the main topic of this study.

### Engineering background

Xincheng gold mine is located in Jincheng Town, Laizhou City, Shandong Province, China. The surface strata in the mining area are simple, mainly in the Fuyang formation of the Archean Jiaodong Group and the Cenozoic Quaternary. Magmatic rocks are widely distributed in the mining area, mainly composed of Linglong monzonitic granite and Guojialing granodiorite distributed in the footwall of the Jiao-Xin main fault plane, and Mesozoic dikes such as lamprophyre and diorite porphyry in the mining area.

**Figure 12:**
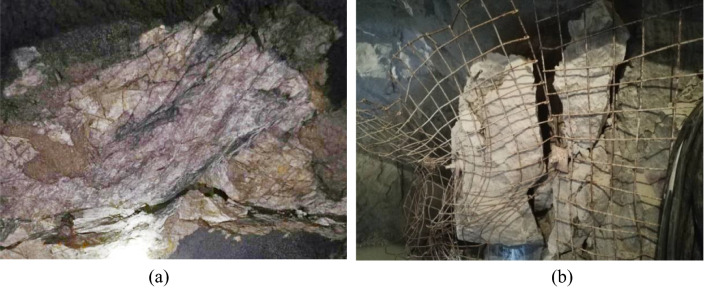
Failure characteristics of roadway surrounding rock in Xincheng Gold Mine. (**a**) Plate cracking of surrounding rock in roadway side, (**b**) the falling off of the surrounding rock after plate cracking.

Xincheng Gold Mine has entered the stage of deep mining, and the mining depth is more than 1200 m. In the process of deep mining, the ground stress is gradually intensified, and the surrounding rock of the roadway will be locally deformed and destroyed with the increase of time, which will lead to the instability and collapse of the surrounding rock of the roadway. On the 1080 m horizontal working face, 60 days after the excavation of the prospecting roadway, the surrounding rock of the roadway appeared local fracture and plate crack failure, as shown in Fig. 12.

### Construction method of numerical model for deep cavern with gradual particle density

**Figure 13:**
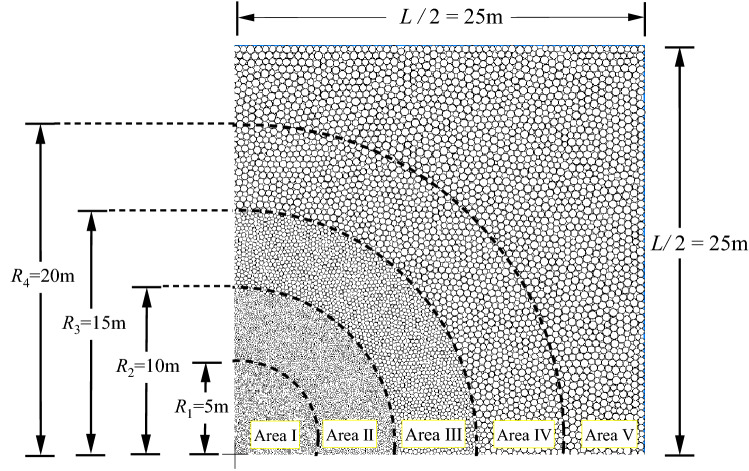
Schematic diagram of gradual change particle density model.

If the large-scale roadway model is established by the traditional modeling method, a large number of particles and long calculation time are needed, and the calculation efficiency is difficult to be guaranteed. Therefore, the variable radius proportional clump model was used to construct the deep cavern numerical model of graded particle density considering the simulation accuracy and calculation efficiency. From the center of the cavern to the outside, the radius of Ball_min_ and Ball_max_ increases step by step, and the particle size ratio of large to small particles is 1.5, and *r*_min_:*r*_max_ = *R*_min_:*R*_max_ = 1.5. Within the radius of *R*_1_ = 5 m, *r*_min_ = 26 mm, *R*_min_ = 60 mm. Within the radius of *R*_2_ = 10 m, *r*_min_ = 50 mm, *R*_min_ = 120 mm. Within the radius of *R*_3_ = 15 m, *r*_min_ = 100 mm, *R*_min_ = 240 mm. Within the radius of *R*_2_ = 20 m, *r*_min_ = 160 mm, *R*_min_ = 300 mm. In the range of *L* = 50 m square shape, *r*_min_ = 180 mm, *R*_min_ = 500 mm. The model generated by the method of numerical calculation of graded particle density is shown in Fig. 13.

Based on the variable radius ratio clump model construction method, a numerical model of the deep cavern with gradual particle density was constructed. In the area close to the project, the particles are denser, and the particles can be enlarged in the far area to reduce the total number of particles. This model can improve the computational efficiency of numerical simulation while meeting the accuracy required by engineering.

### Numerical calculation model size and boundary conditions

**Figure 14:**
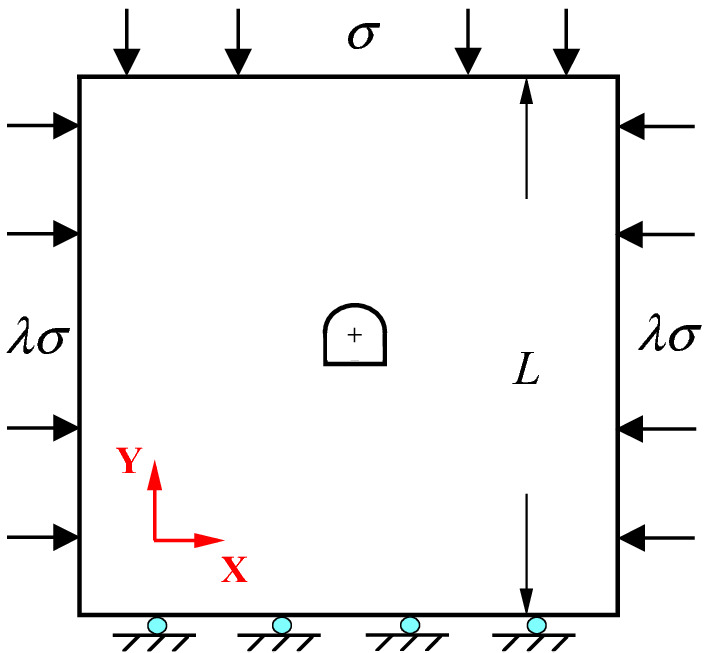
Diagram of numerical calculation model.

Taking the roadway of Xincheng Gold Mine as the engineering background, a two-dimensional numerical calculation model was established. The boundary of the model is the square with a side length *L* = 50.0 m. The radius of the semicircle of the upper part of the horseshoe tunnel cavern is *R* = 3.0 m, the height of the lower part of the rectangle is *H* = 1.5 m, the lateral pressure coefficient *c* = 0.5, and the initial rock stress *σ* = 100 MPa. A schematic diagram of the numerical calculation model is shown in Fig. 14.

To simulate the stress state in the area where the high-stress tunnel is located, the initial stress state was applied to the model by wall servo. The servo mechanism establishes a relationship between velocity and stress, where velocity is a function of pressure. The contact force on the wall needs to be monitored in the system, and when the target force is not reached, the target stress is achieved by applying velocity to adjust the position of the wall to achieve a squeeze or release effect.

### Model parameters

Based on the sensitivity analysis of mesoscopic parameters and the agreement between the numerical simulation of uniaxial compressive strength test and Brazilian disc splitting test and the indoor test results, the parallel bond model was still used to calculate the mechanical parameters of high-stress tunnel model as shown in Table 8.

**Table 8:** Mechanical parameters of surrounding rock in deep cavern.

Parameters	Value
Ball stiffness ratio *k*_*n*_*/k*_*s*_	2.0
Ball friction coefficient *µ*	0.2
Parallel bond modulus E*/Gpa	2.0
Particle stiffness ratio *k*_n_*/k_s_*	2.5
Parallel bond tensile strength *σ*_*b*_/Mpa	32
Parallel bond cohesion *c*_*b*_/Mpa	24
Particle friction coefficient µ*	0.2
Friction angle *ƒ*/°	32

### Evolution law of deep surrounding rock local instability

After the tunnel excavation, due to the excavation unloading and the pressure exerted by the overlying rock and soil, the surrounding rock breaks and shows signs of instability and failure. In order to study the failure mechanism of high-stress surrounding rock, the deep cavern numerical model with gradual particle density was calculated and solved, and the microscopic characteristic indexes of roadway surrounding rock were analyzed, including stress field and microfracture field.

#### Evolution law of stress field

**Figure 15:**
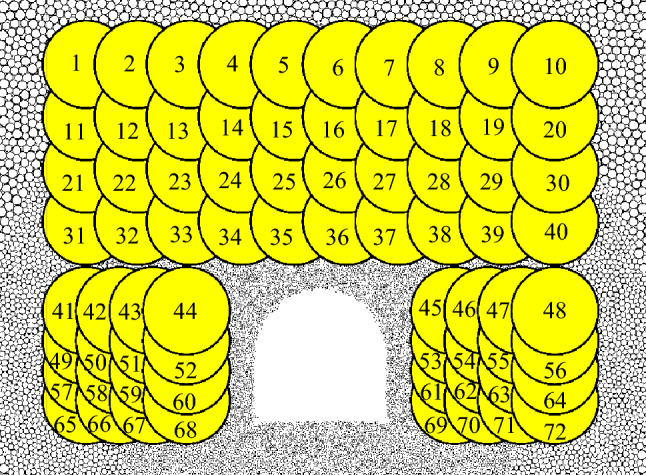
Diagram measuring circle.

In order to understand the failure mechanism of excavation face, the deformation and stress of rock mass were monitored in the process of simulation, and the command of measuring circle was used. This command can measure the stress, displacement, voidage, rotation angle and other parameters of the particles defined in the measuring circle. No. 1-72 measuring circles were set on the roadway vault and the two sides, and the arrangement is shown in Fig. 15.

**Figure. 16:**
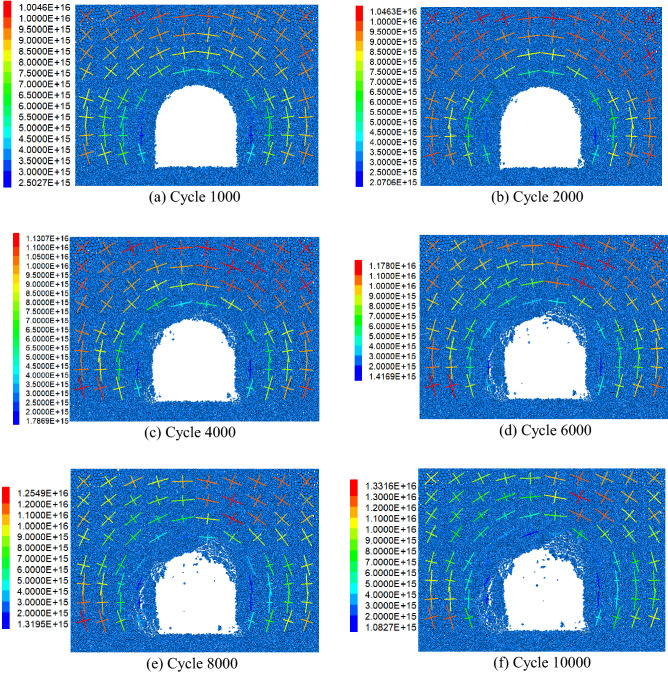
Stress distribution after simulated excavation.

In the process of roadway excavation, the stress field distribution can be used to express the characteristics of stress change, as shown in Fig. 16. The stress field consists of the principal stress values and their principal directions in each measuring circle, where the length of the line represents the relative magnitude of the principal stress.

Figure 16 illustrates that the direction of the large principal stress was approximately parallel to the empty face of the roadway, the direction of the small principal stress was approximately perpendicular to the empty face of the roadway. The overall stress state of the surrounding rock of the roadway was arched.

After 1000 steps of simulated excavation, the maximum principal stress increased gradually from near to far from the open face of the roadway, and the stress state of the surrounding rock far exceeded the strength of the surrounding rock, which was easy to cause damage, as shown in Fig. 16a.

After 2000 steps of simulated excavation, the maximum principal stress of the two sides of the roadway showed a decreasing trend, and there was a fracture zone in the arch foot and vault of the roadway, as shown in Fig. 16b.

After 4000 steps of simulated excavation, the maximum principal stress of the two sides of the roadway continued to decrease, and the fracture zone of the arch foot and vault of the roadway increased, as shown in Fig. 16c.

After 6000 steps of simulated excavation, the maximum principal stress of the two sides of the roadway continued to decrease, and the maximum principal stress of the outside of the left arch foot and the upper part of the right arch increased obviously, as shown in Fig. 16d.

After 8000 steps of simulated excavation, the rock mass in the fracture zone of the left wall and the right vault of the roadway was relatively broken, and the maximum principal stress in the upper part of the right vault continued to increase, as shown in Fig. 16e.

After 10,000 steps of simulated excavation, the maximum principal stress of the two sides of the roadway and the vault was the smallest, and the maximum principal stress of the upper part of the right vault was the largest, as shown in Fig. 16f.

#### Local fracture law

**Figure. 17:**
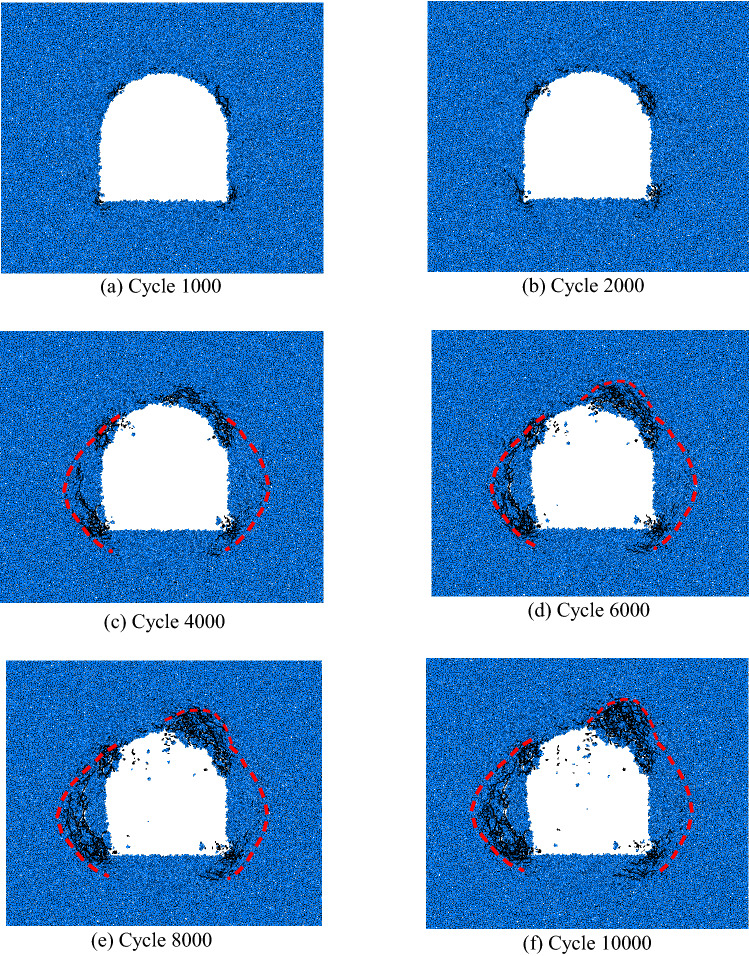
Expansion law of crack after simulated excavation.

Local fracture law is the main manifestation of the progressive failure of surrounding rock. The initiation, propagation, aggregation and interaction of microcracks reduce the mechanical properties of rock mass, and finally form macroscopic cracks. When the interparticle stress is greater than the bond strength, the bond is destroyed and microcracks are formed. The deformation law of the simulated crack after excavation is shown in Fig. 17.

After 1000 steps of simulated excavation, cracks first appeared at the two arch feet and the vault, as shown in Fig. 17a.

After 2000 steps of simulated excavation, the microcracks in the arch foot and vault continued to develop, and the cracks gradually increased, propagated and accumulated, as shown in Fig. 17b.

After 4000 steps of simulated excavation, the triangular fracture zone began to appear in the left wall of the roadway, and the thickness of the fracture area was about 0.5*R*. The cracks in the right vault increased and gathered, and began to produce scattered particles, as shown in Fig. 17c.

After 6000 steps of simulated excavation, the triangular fracture zone in the left side wall of the roadway expanded, and the thickness of the fracture zone was about 0.6*R*. The cracks in the right arch continued to increase and gather obviously, as shown in Fig. 17d.

After 8000 steps of simulated excavation, the scattered particles increased, the triangular fracture zone in the left wall of the roadway continued to expand, the thickness of the fracture area was about 0.8R, the rock mass in the fracture zone was relatively broken, and the boundary of the rupture area of the right vault was clear. and has gradually moved away from the cavern outline and moved inward, as shown in Fig. 17e.

After 10,000 steps of simulated excavation, an obvious shear zone appeared along the boundary of the triangular fracture zone in the left wall. The distribution of the crack propagation zone was concentrated in the triangle area, and the thickness of the fracture zone was about 1.0*R*. The thickness of the fracture zone of the right vault was about 0.8*R*, and the crack boundary of the fracture zone was relatively clear, as shown in Fig. 17f.

**Figure 18:**
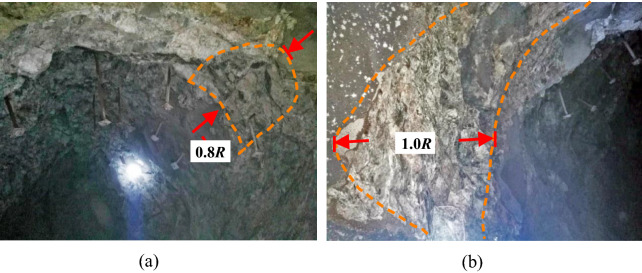
Failure characteristics of surrounding rock of deep roadway. (**a**) shear failure of surrounding rock in roadway vault, (**b**) local failure of roadway side.

Although the surrounding rock of the roadway in Xincheng Gold Mine is mainly relatively hard granite, the surrounding rock of the roadway appeared the phenomenon of local instability and fracture under the condition of high ground stress. Especially in the roadway vault and helper, there is an obvious shear failure area, as shown in Fig. 18.

The numerical simulation results showed that the failure form of the surrounding rock was mainly brittle failure, and the rupture area of the right vault of the roadway was obvious. There was an approximate triangular fracture zone on the left wall of the roadway, and the failure area did not extend to the deep part of the surrounding rock of the roadway. In the actual project, the shape and depth of the fracture zone of the right vault and left side wall of the roadway were basically the same as the results of numerical simulation for a period of time after construction. The numerical calculation model of gradual particle density proposed in this paper can reasonably reflect the local instability and fracture characteristics of the surrounding rock of high-stress cavern, and the calculation results can provide reference for the actual roadway construction.

## Conclusion

1. A construction method of variable radius proportional clump model was proposed with particle flow method. Comparison of the UCS/TS of ball and clump particle structures with different particle size variations and meso-mechanical parameters revealed that, it was slightly influenced by meso-mechanical parameters. Ball model was also slightly influenced by particle size but clump model was highly influenced by particle size and proportion.
2. Using the proposed variable particle size ratio clump model, simulation and laboratory compressive and tensile fracture modes and strength curves were compared. The obtained results revealed good compliance between laboratory and simulation findings. When constructing particle models for other rock types, clump particles could be adjusted to reflect different compressive and tensile mechanical properties.
3. Based on the proposed variable particle size ratio clump model construction method, the numerical model of deep cavern with gradual particle density was constructed. Comparing the numerical simulation results with the engineering practice, the damage phenomenon of the roadway vault and sidewall was very close. The gradual particle density numerical model can reasonably reflect the failure law of the surrounding rock in high-stress cavern.

## Data Availability

The datasets used or analyzed during the current study are available from the corresponding author on reasonable request.
